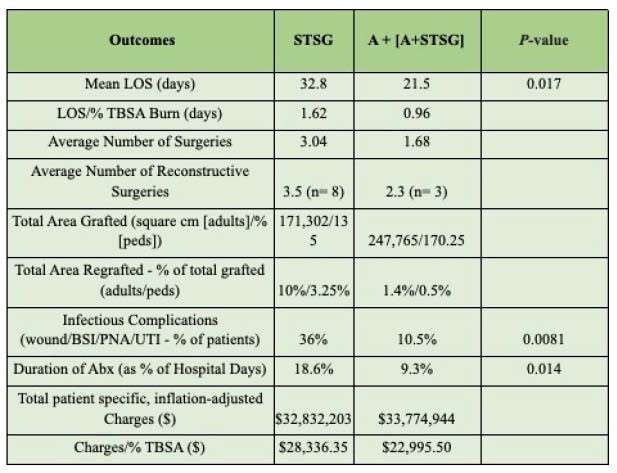# 298 Use of Autologous Skin Cell Suspension Improves Clinical Outcomes and Patient Charges in Large Burns

**DOI:** 10.1093/jbcr/irad045.273

**Published:** 2023-08-29

**Authors:** Djoni Elkady, Beverly Beaucock, Anjay Khandelwal, Richard B Lou

**Affiliations:** Loyola University Stritch School of Medicine, Maywood, Illinois; Akron Children’s Hospital, Akron, Ohio; Akron Children’s Hospital, Akron, Ohio; Akron Children’s Hospital, Akron, Ohio

## Abstract

**Introduction:**

The use of autologous skin cell suspension (ASCS) is an adjunct to the standard of care for definitive wound closure for burn patients. Several published predictive models have indicated theoretical reductions in length of stay (LOS) and hospital costs. There are no studies comparing a single institution’s clinical and surgical results, and patient specific, inflation adjusted, cost/charge data prior to and after utilizing ASCS.

**Methods:**

A retrospective study was conducted of all patients with >10% TBSA burn from January 2017 to July 2022. The use of ASCS was standardized in January 2019. Demographics, co-morbidities and clinical/surgical outcomes (LOS, wound infections, other infectious complications, square cm [adults] or % TBSA [pediatrics] grafted and re-grafted, number of surgeries, number of reconstructive surgeries as well as use and duration of antibiotics were recorded. Patient specific, annual institutional inflation adjusted charges as a surrogate for cost of care were analyzed. Comparative groups included all patients with >10% TBSA burns treated without ASCS (“Pre”) versus all patients treated with ASCS (“Post”) as well as a post-hoc analysis of patients treated surgically with either split thickness skin graft (“STSG”) versus ASCS, either alone (“A”) or in conjunction with STSG (“A+STSG”). For comparative analysis all patients treated with ASCS patients started in January 2019 were included when a standard approach was created. Statistical analysis including regression modeling was performed.

**Results:**

168 met inclusion criteria of which 92 patients were in the “Pre” group (66 treated with STSG and 26 conservatively without surgery) and 76 patients were in the “Post” group (either A [12] or A+STSG [64]). Significant demographic differences were % TBSA and history of hepatic disease (both higher in “Post” group) - see table. Patients in the “Post” group had decreased LOS and LOS/%TBSA, number of surgeries, infectious complications, use of and duration of antibiotics and charges/%TBSA. Sub group analysis of “STSG” versus “A” + “A+STSG” (all patients undergoing surgery) again demonstrated the above findings as well as decreased percentage of TBSA requiring re-grafting (STSG: 10% vs A/A+STSG: 1.4% in adults and STSG: 3.25% vs A/A+STSG: 0.5% in pediatrics) - see table. On average, there was a decrease of $5,340.85/%TBSA in the “A” +”A+STSG” versus the “STSG” group.

**Conclusions:**

In a single institution,, the addition of ASCS for coverage of burns >10% TBSA compared to SOC resulted in improved clinical outcomes and cost of care (decreased LOS, infectious complications, duration of antibiotic usage and re-grafting rates as well as decreased charges/%TBSA burn). This is the first study to demonstrate actual differences in multiple clinical/surgical outcomes and patient charges.

**Applicability of Research to Practice:**

The addition of ASCS in the surgical management of burn patients may improve clinical outcomes and lead to decreased cost of care.